# The efficacy of low‐power cumulative high‐intensity focused ultrasound treatment for recurrent desmoid tumor

**DOI:** 10.1002/cam4.4573

**Published:** 2022-03-11

**Authors:** Xian Zhong, Xiaoye Hu, Peng Zhao, Yuebing Wang, Xue Feng Fang, Jiayi Shen, Hong Shen, Ying Yuan

**Affiliations:** ^1^ Department of Medical Oncology, Key Laboratory of Cancer Prevention and Intervention, National Ministry of Education, Key Laboratory of Molecular Biology in Medical Sciences, Second Affiliated Hospital Zhejiang University School of Medicine Hangzhou Zhejiang China; ^2^ College of Metrology and Measurement Engineering China Jiliang University Hangzhou China; ^3^ Zhejiang University‐University of Edinburgh (ZJU‐UOE) Institute, Zhejiang University School of Medicine Haining China

**Keywords:** aggressive fibromatosis, desmoid tumor, efficacy, high‐intensity focused ultrasound, low‐power cumulative HIFU

## Abstract

**Background:**

Desmoid tumors are rare neoplasms that are locally invasive. However, optimal treatment strategies for recurrent desmoid tumors remain controversial. High‐intensity focused ultrasound (HIFU) has been reported as a noninvasive modality for treating recurrent desmoid tumors. However, its efficacy against massive desmoid tumors or those with complex anatomies remains unclear.

**Methods:**

We developed a new therapeutic strategy called low‐power cumulative HIFU and applied it to treat recurrent desmoid tumors.

**Results:**

We retrospectively collected data from 91 patients with recurrent desmoid tumors who underwent low‐power cumulative HIFU treatment after surgical treatment failure. The mean ablation proportion of the HIFU treatment was 69.5%, and the objective response rate was 47.3%. The 5‐year estimated progression‐free survival rate for these patients was 69.3%.

**Conclusion:**

Low‐power cumulative HIFU treatment could achieve significant efficacy and long‐term control of recurrent desmoid tumors.

## Graphical abstract

Desmoid tumor, also called aggressive fibromatosis, is a rare and locally invasive soft tissue neoplasms. We developed a new strategy called low‐power cumulative high intensity focused ultrasound resulting in a precise and localized ablation for recurrent desmoid tumors. In our study with the largest sample size, this novel treatment showed great efficacy and long‐time control, even cure for some patients.

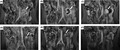

## 1 INTRODUCTION

Desmoid tumors are rare soft tissue lesions with local invasiveness, primarily affecting young patients. The incidence is approximately five patients per million people per year.^1^ Desmoid tumors can be roughly classified into two subtypes: sporadic desmoid tumors and hereditary desmoid tumors. Typically, hereditary desmoid tumors are accompanied by familial adenomatous polyposis or Gardner syndrome.^2^

Although several effective treatments for recurrent desmoid tumors have been reported, the optimal regimen remains controversial.^3^ Close observation may be the first choice of treatment for asymptomatic patients. However, treatment may be pursued in patients with progressive disease, symptoms, and the risk of adjacent structure involvement. Given that the proximity of adjacent vital structures may preclude appropriate surgery for some recurrent desmoid tumors, wide resection with sufficient negative margins might be markedly difficult to achieve. Radiotherapy could be a better strategy than surgical treatment, as the complex anatomy would interfere with surgical interventions,^4^, ^5^ although radiotherapy can cause complications such as skin and nerve toxicity in pediatric and young adult patients.^6^ Recently, considerable attention has been paid to systemic therapies, including hormones, non‐steroidal anti‐inflammatory drugs, chemotherapy, and tyrosine kinase inhibitors.^2^, ^7^, ^8^ However, these therapies may be inadequate to control the development of desmoid tumors.

Recent studies have also revealed that high‐intensity focused ultrasound (HIFU) could be a feasible treatment for several types of tumors, such as thyroid nodules, liver cancer, and pancreatic cancer.^9^, ^10^, ^11^ Under real‐time imaging guidance, HIFU could precisely ablate the targeted area with thermal coagulation necrosis. In addition, previous studies have indicated that HIFU could be a non‐invasive and effective treatment for recurrent desmoid tumors. However, for desmoid tumors with complex anatomies, such as intra‐abdominal or cervical desmoid tumors, clinicians should be cautious when performing HIFU treatment. Accordingly, we adjusted HIFU and used low‐power input plus emission. In this strategy, the speed of heat diffusion and accumulation is relatively slow, affording higher stability, thus decreasing damage to the adjacent normal tissue. Our previous study has demonstrated encouraging outcomes of ultrasound‐guided low‐power cumulative HIFU treatment in four patients with recurrent desmoid tumors.^12^ To date, we have treated more than 100 patients. Herein, we describe our experience with ultrasound‐guided low‐power cumulative HIFU ablation treatment in patients with recurrent desmoid tumors and present long‐term follow‐up results, with the largest sample size.

## 2 MATERIALS AND METHODS

Data from 91 patients with recurrent desmoid tumors were retrospectively collected between June 2011 and July 2019 at the Department of Surgery Oncology and Medical Oncology, the Second Affiliated Hospital, Zhejiang University, School of Medicine. All patients fulfilled the following inclusion criteria: (1) histological diagnosis of desmoid tumor, (2) recurrent tumor after surgery, (3) tumor visible on ultrasound, and (4) Karnofsky performance status >50. The main exclusion criteria were (1) non‐eligibility for general anesthesia and (2) extensive scarring along the acoustic path. The present study was approved by the ethics committee of the Second Affiliated Hospital, Zhejiang University, School of Medicine.

HIFU treatment with real‐time ultrasound guidance was performed under anesthesia using the FEP‐BY02 system (Beijing Yuande Biomedical Engineering) and guided by a diagnostic ultrasound device (Logic 5 or 9, GE Healthcare), as described in our previous study.^13^ The parameters of HIFU treatment were as follows: ultrasonic power 100–300 W (input power varied depending on the depth of the tumor); unit transmission time or pulse length (T1)/intermission time or pulse repetition period (T2) 990/10 ms; 40 transmissions per therapeutic point with a distance of 2 mm between adjacent therapeutic points; treatment of each unit (5 therapeutic points) for 200 s with an interval of 2 min between each unit, spacing of 5 mm between adjacent treatment slices; total emission lasting <90 min. Contrast‐enhanced ultrasonography was employed for the preliminary evaluation of HIFU ablation during treatment. The typical treatment was performed for 1–2 h, depending on the tumor size and location. Large tumors near vital organs or structures necessitate prolonged treatment times. The planned ablation area should envelop the entire tumor with a margin of at least 1 cm. In general, the intervention was fractionated over several tumor areas. For a tumor size <10 cm, the ablation area was initially planned to be 60–80% of the tumor volume. For a tumor size >10 cm, the ablation area was initially planned to be 30–50%. After approximately 1 month, HIFU ablation was performed again, based on MR examination, and repeated monthly to achieve a satisfactory effect. During the follow‐up, the occurrence of complications was carefully documented.

The tumor lesions were evaluated using magnetic resonance imaging (MRI). Enhanced areas on MRI were considered viable tumors, whereas lesions without enhancement were assumed to be necrotic tissue. The ablation efficacy was defined as the proportion of necrotic area within 1 month after the end of all HIFU treatments. Follow‐up intervals were 3–6 months with evaluation by computed tomography or MRI. Follow‐up was terminated on 10 October 2020. Tumor response was assessed according to the Response Evaluation Criteria in Solid Tumors (RECIST 1.1).^14^ Given that desmoid tumors are slow‐growing, the duration of stable disease (SD) is defined as two follow‐up intervals.

Statistical analyses were performed using SPSS version 20.0 (IBM SPSS). Progression‐free survival (PFS) time was measured from the date of first treatment to the date of disease progression or death. PFS estimates were analyzed using the Kaplan–Meier method.

## 3 RESULTS

A total of 91 patients with 122 desmoid tumors underwent low‐power cumulative HIFU treatment after surgical treatment failure. The median age of these patients was 28 years (range, 14–69 years). The mean tumor diameter was 9.4 cm (range: 1.9–32.7 cm), of which 44 (36%) patients had tumors ˃10 cm in diameter. Before HIFU treatment, 47 patients had undergone two or more operations and failed surgery. Among them, one patient underwent eight surgical interventions for recurrent tumors, which were controlled by low‐power cumulative HIFU treatment without progression for ˃ 9 years. Table 1 summarizes the clinical characteristics of the patients.

**TABLE 1:** Clinical characteristics of patients (91 patients with 122 tumor lesions)

Sex	
Male	21/91 (23.1%)
Female	70/91 (76.9%)
Age (median)	28 (range, 14–69 years)
Surgery before HIFU	
1 time	44/91 (48.3%)
2 times	27/91 (29.7%)
>2 times	20/91 (22.0%)
Tumor size (mean)	9.4 (1.9–32.7 cm)
<10 cm	78/122 (64%)
10–20 cm	36/122 (29.5%)
>20 cm	8/122 (6.5%)

Abbreviation: HIFU, high‐intensity focused ultrasound.

**FIGURE 1:**
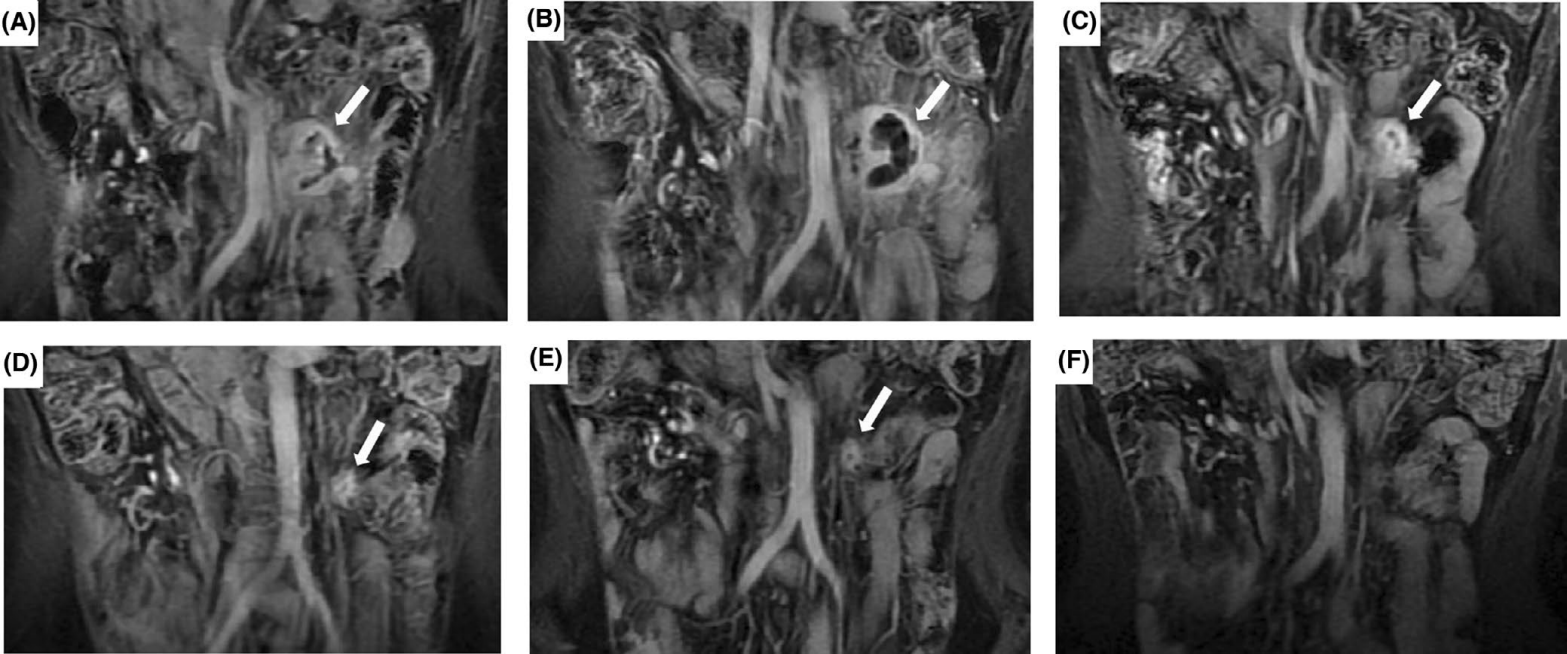
A recurrent desmoid tumor in a 46‐year‐old woman was carefully evaluated by MRI. (A). MRI images show intra‐abdominal recurrence before HIFU treatment; (B). Significant necrosis can be observed after 3 days; (C). Tumor shrinkage can be observed after 5 months; (D). MRI images after 12 months; (E). MRI images after 20 months; (F). The complete remission of the recurrent desmoid tumor can be observed after 41 months of follow‐up after HIFU treatment. MRI, magnetic resonance imaging; HIFU, high‐intensity focused ultrasound

**FIGURE 2:**
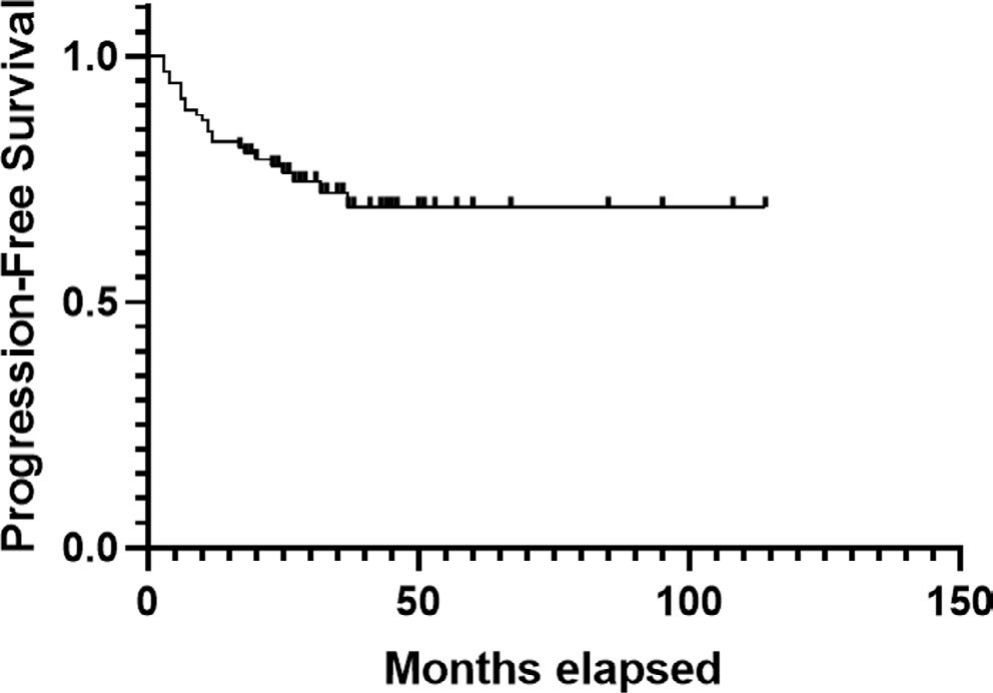
Kaplan–Meier plots displaying progression‐free survival

The ablation efficacy was evaluated using MRI (Figure 1). The mean ablated proportion of these tumors was 69.5% (95% CI: 64.6%–74.3%). In addition, 15 tumors were 100% ablated by low‐power cumulative HIFU treatments. During follow‐up (mean follow‐up time: 28 months; range: 17–114 months), significant tumor shrinkage was observed in most patients within 6 months to 1 year. The objective response rate was 47.3% (43/91), and the disease control rate was 96.7% (88/91) (Table 2). Complete response (CR) was observed in 12 patients (median duration: 30 months; range: 5–90 months), of which two patients had two lesions. Three patients showed disease progression after low‐power cumulative HIFU treatments; one patient, who previously received multiple treatments (two surgical interventions, radiotherapy, cryotherapy, radioactive seed implantation, and another operation with recurrent intra‐abdominal desmoid tumor lesion) accepted HIFU treatment, but tumor progression was detected in 3 months, followed by death due to bowel obstruction 2 months later. Except for this patient, all participants in the present study survived. The Kaplan–Meier curve of PFS time is shown in Figure 2. The 5‐year estimated PFS rate was 69.3%.

**TABLE 2:** Results of low‐power cumulative HIFU treatment

Ablation efficacy (mean)	69.5%
100% lesion ablation	15/122 (12.3%)
Patient tumor response	
CR	12/91 (13.2%)
PR	31/91 (34.1%)
SD	45/91 (49.4%)
PD	3/91 (3.3%)
Median PFS	Not reached
Median OS	Not reached

Abbreviations: CR, complete response; HIFU, high‐intensity focused ultrasound; OS, overall survival; PD, progressive disease; PFS, progression‐free survival; PR, partial response; SD, stable disease.

The main adverse effects of low‐power cumulative HIFU treatment are skin burns, nerve injury, and perforation. Twenty patients experienced treatment‐related skin burns. Among them, 18 patients experienced grade I/II skin burns, while 2 patients experienced grade III skin burns, warranting a longer duration of recovery. One patient who underwent four surgical treatment failures and one radiation procedure exhibited grade III skin burn owing to surgical scars on the targeted regions, requiring 4 months for recovery with medications. A total of 10 patients experienced nerve injury, including 8 patients who experienced mild neurological damage and recovered within 3 months and 2 patients with severe neurological damage. Two patients with post‐peritoneal recurrent tumors infiltrating the intestine developed intestinal perforation and recovered from surgery. Of the 91 patients, 1 patient had severe infection with abscess formation and recovered after drainage. No accumulated dose limitation was found for low‐power cumulative HIFU in the present study.

## 4 DISCUSSION

Desmoid tumors are slow‐growing, non‐metastasizing tumors with a highly invasive capacity, rendering such tumors suitable for local treatments, including local thermal therapy and radiotherapy, in the case of surgical treatment failure. HIFU ablation treatment is an extracorporeal conformal treatment that induces coagulative necrosis of tumor tissue by rapid thermal effects and cavitation.^15^ In recent decades, HIFU ablation has shown remarkable efficacy and safety in local treatments. Several studies have reported that HIFU ablation could be a suitable approach for recurrent desmoid tumors, presenting an ablation efficacy that ranged between 50 and 92.5%.^16^, ^17^, ^18^, ^19^ The mean decrease in tumor size was reportedly 36%.^20^ Twelve after treatment, the tumor regression rate was 58.2%.^21^ However, most studies have merely reported the efficacy and safety of HIFU ablation for extra‐abdominal desmoid tumors. For desmoid tumors with complex anatomies, such as intra‐abdominal or cervical desmoid tumors, it is difficult to achieve maximum efficacy with HIFU treatment without detrimental complications, given its potential damage to normal tissues. At our medical center, low‐power cumulative HIFU is recognized as a well‐established technique for desmoid tumor treatment. As we previously reported, patients with recurrent desmoid tumors who underwent low‐power cumulative HIFU were more likely to achieve enhanced tumor ablation efficacy. This indicates that continuous low‐power cumulative HIFU treatment would achieve superior efficacy and reduce harm to patients when compared with the traditional HIFU treatment. In addition, with the guidance of real‐time ultrasound imaging, the low‐power cumulative HIFU‐induced hyperthermia would be more precise, thus presenting fewer deleterious complications. Hence, patients with massive desmoid tumors (e.g., tumors ˃10 cm in diameter) or patients with desmoid tumors accompanied by complex anatomies would benefit from this treatment. Consistent with this concept, the tumor ablation rate of low‐power cumulative HIFU treatment for desmoid tumors was 69.5% in the cohort at our medical center.

During follow‐up, 12 of the 91 patients with recurrent desmoid tumors showed CR following treatment. The longest follow‐up duration after achieving complete remission was 8 years. Our results suggest that low‐power cumulative HIFU could potentially cure some patients with recurrent desmoid tumors. Although low‐power cumulative HIFU ablation failed to accomplish complete tumor ablation in all recurrent tumors, a significant debulking effect was achieved. As documented in our hospital, the best objective response rate was 47.3%, and the disease control rate was 96.7%.

Patients with recurrent desmoid tumors accompanied by complex anatomies, especially those located in the intra‐abdominal and neck regions, can scarcely achieve complete or high ablation. However, we observed that the low‐power cumulative HIFU treatment could control tumor growth while minimizing side effects in these patients. Interestingly, we also observed that some patients with desmoid tumors initially showed minor HIFU ablation responses, while their desmoid tumor volume gradually decreased within 2 years after treatment. In such patients, “watch and wait” could be an effective strategy after HIFU treatment when the tumor fails to shrink significantly.

The side effects of HIFU treatment, such as skin burns, nerve injury, and intestinal perforation, have been previously reported. In the present study, the frequencies of skin burns and nerve injuries were comparable with those observed in other studies using HIFU treatment for desmoid tumors. As intra‐abdominal desmoid tumors were also included in the present study, as described above, two patients with intra‐abdominal desmoid tumors experienced intestinal perforation during the early phase of this study. To achieve satisfactory ablation results and simultaneously reduce potential complications, the time interval between HIFU treatments should be adjusted according to the ablation area and anatomic structures on MRI. Multiple ablations are required for patients with massive desmoid tumors to prevent prolonged anesthesia and excessive heat accumulation. If there is extensive scarring in the proposed pathway of the HIFU beam, careful attention should be paid to prevent irreversible skin burns.

In conclusion, the present study demonstrates that low‐power cumulative HIFU treatment of recurrent desmoid tumors can feasibly reduce viable tumor volumes and control tumor growth over a prolonged period. However, multicenter, prospective studies are needed to confirm these findings.

## CONFLICT OF INTEREST

The authors declare no conflict of interest.

## AUTHOR CONTRIBUTIONS

Conceptualization, Xian Zhong, Xiaoye Hu and Hong Shen; Investigation, Xian Zhong, Peng Zhao and Yuebing Wang; Methodology, Peng Zhao, Yuebing Wang and Xiaoye Hu; Supervision, Ying Yuan; Writing‐original draft, Xian Zhong; Writing‐review & editing, XueFeng Fang, Jiayi Shen and Hong Shen.

## ETHICS STATEMENT

The study was approved by the ethics committee of the Second Affiliated Hospital, Zhejiang University, School of Medicine, with ethical approval code 2020–831. Informed consent was obtained from each patient.

## Data Availability

The data that support the findings of this study are available from the corresponding author upon reasonable request.
